# Mobile Electronic Medical Records Promote Workflow: Physicians’ Perspective From a Survey

**DOI:** 10.2196/mhealth.5464

**Published:** 2016-06-06

**Authors:** Julian Duhm, Robert Fleischmann, Sein Schmidt, Hagen Hupperts, Stephan A Brandt

**Affiliations:** ^1^ Vision and Motor System Research Group Department of Neurology Charité - Universitätsmedizin Berlin Berlin Germany; ^2^ Clinical Research Unit Berlin Institute of Health Berlin Germany; ^3^ Department of Information Technology Charité - Universitätsmedizin Berlin Berlin Germany

**Keywords:** tablet PC, electronic health record, usability, health services, inpatient care

## Abstract

**Background:**

As a result of demographic changes, physicians are required to deliver needed services with limited resources. Research suggests that tablet PCs with access to patient data may streamline clinical workflow. A recent study found tablets with mobile electronic medical records (EMRs) can facilitate data retrieval and produce time savings across the clinical routine within hospital settings. However, the reasons for these time savings, including details on how tablets were being used, remain unclear. The same applies to physicians’ perceptions of this tool within an inpatient setting.

**Objective:**

This study examined physicians’ perception of tablets with EMRs in an inpatient setting. The rationale was to identify both subjective and objective factors that impacted the successful implementation and use of tablets running an EMR.

**Methods:**

We developed a 57-item survey questionnaire designed to examine users’ perception of and attitude toward tablets, which was administered to 14 participating physicians following 7 weeks of tablet use. Five participants volunteered to participate in a second study that investigated physicians’ patterns of tablet use within the EMR environment by digitally tracking and storing usage behavior. Statistical analyses of questionnaire results included mean values with their bootstrapped 95% confidence intervals and multivariate analysis of variance to identify predictors of tablet use.

**Results:**

Physicians reported high degrees of satisfaction with the tablets. There was a general consensus among physicians that tablet use streamlined clinical workflow through optimized data retrieval (rated 0.69, 0.23-1.15 points better than control) and improved communication with patients and other physicians (rated 0.85, 0.54-1.15 and 0.77, 0.38-1.15 points better than control, respectively). Age (F3,11=3.54, *P*=.04), occupational group (F1,11=7.17, *P*=.04), and attitude toward novel technologies (F1,11=10.54, *P*=.02) predicted physicians’ satisfaction with the devices and their motivation regarding their further use. Tracking data yielded that only a few of the available functions were used frequently.

**Conclusions:**

Although tablet PCs were consistently perceived as beneficial, several factors contributed to the fact that their full potential was not fully exploited. Training in functionality and providing a reliable infrastructure might foster successful tablet implementation.

## Introduction

An increasing average life expectancy paralleled by a declining birth rate has ushered in a constant demographic change in industrialized nations [1]. These processes have significantly affected health care service providers. In Germany, the most tangible consequences of the aging population are a 28% increase of cases treated in hospitals since 1991 [2] and a 46% decrease in length of stay per patient [3]. Within the same period, hospitals have registered a dramatic increase in the inpatient treatment of patients aged 50 years or older [2]. Clear evidence indicates that the number of diagnoses per patient ascends with age [4], meaning that older patients often require more extensive and costly treatment than the younger population [5]. As a direct consequence, expenditures for health state insurance in Germany rose by approximately 30% over the last decade [6]. Although experts expect this drift to progress, similar trends have been observed in other industrialized countries [7].

As a result, health care service providers find themselves in a position where they have to maintain good quality of services while dealing with sicker patients that often require more complex, and more time-consuming, treatment and diagnostic procedures. The clinical routine has thus become denser and involves handling larger amounts of data in less time. This underpins the need to optimize data handling within the clinical environment to enhance practitioners’ efficiency. In addition, a growing gap in physician supply has been forecasted for at least one other Western nation [8]. This further highlights the need for streamlining clinical workflow. In line with this notion, physicians at German hospitals spend up to one-third of their average daily labor time on clinical documentation [9]. Therefore, this area in particular may benefit from workflow enhancements. Recent research provides solid evidence that quality and efficiency of health care delivery can be improved through the use of digital information systems [10]. Hence, it is unsurprising that many hospitals have already replaced traditional paper charts with electronic information systems to grant more efficient and less time-consuming data handling [11]. A solid base of evidence indicates that electronic medical records (EMRs) yield both process and structural benefits [12]. They can further promote availability of and access to patient data [13], which is of great importance considering that access to these data is often crucial in making medical decisions [14]. Although mobile EMRs provide location-independent access to patient data in real time [15], it has been asserted that modern tablet PCs may be appropriate hardware in this context [16]. Recent research suggests that such devices possess several features that make them particularly suitable for the clinical environment. These include large screens that offer convenient access to graphical content, long battery life, a high degree of portability, and sufficient storage capacity [17]. Positive patient attitude toward tablet use by doctors [18], easy disinfection, and the availability of a broad selection of medical apps make the devices even more attractive for professional use within medical settings [16]. In summary, tablets unarguably offer an appealing potential for clinical use.

However, until recently there were no studies investigating the impact of tablets with EMRs on the clinical routine within an inpatient setting. In a first attempt to shed light on this current issue, we conducted a study that aimed to examine potential benefits of tablets with mobile EMRs on a hospital ward [19]. In this context, we explored quantitative effects of tablet use as an extension of the established gold standard (paper chart and trolley with laptop running an EMR) on labor time in comparison to exclusive use of the existing gold standard. Results showed that tablet use led to significant time savings during the preparation and postprocessing of ward rounds. We also found that checking a medical record was significantly faster in the presence of a tablet, which in turn led to a significant increase in doctor’s time spent at the bedside. Yet, the underlying mechanisms of the obtained time savings remained unclear.

In addition to quantitative improvements as outlined previously, early qualitative studies report high degrees of satisfaction with mobile clinical information systems among physicians [20]. However, these studies were conducted before the tablet PC era. Research investigating physicians’ perceptions of tablets in the clinical environment thus remains sparse. Anderson et al [16] examined physicians’ perception of tablets in private practice settings. They found that physicians responded generally positively to the devices. Findings from a second qualitative study that was conducted in an emergency department suggest that tablet use can potentially streamline clinical workflow and improve physician-patient interaction [21]. These results are promising overall. However, none of these studies were conducted within an inpatient setting and, importantly, subjective reports (eg, through questionnaires) were rarely correlated with changes in objective measures. In order to fill this gap in the literature and to better understand the underlying causes for the significant time savings observed in our previous study [19], we gathered data through semistructured questionnaires. We further electronically tracked patterns of tablet use by participating physicians to gain a better understanding of how the tablets were utilized during the clinical work day. Our aim was to get a clear grasp of what features exactly were perceived as beneficial by physicians.

## Methods

### Participants

The study was conducted at the department of neurology at the Charité University Hospital in Berlin. Nine resident (2 female, 7 male) and five staff neurologists (all male) participated in the study. Two participants were younger than 30 years of age, seven were aged between 30 and 39 years, four were aged between 40 and 49 years, and one was older than 50 years. The main sampling criterion was to select physicians who regularly engaged with the clinical routine. This criterion was met by all participants. Physicians were informed that they would be required to provide feedback regarding their practical experience with the tablets following the study period. A total of five participants agreed to participate in the electronic data tracking. All participants gave informed verbal consent before completing the questionnaire. No information regarding hypotheses was disclosed at this point. Data collection was fully confidential. Participants were told not to use their tablet in case they felt that this would hinder the delivery of health service in any conceivable sense (eg, in the case of an emergency). Participants were free to withdraw from the study at any point. However, none of the participants withdrew. The study received approval from the Ethical Review Board of the Charité Teaching Hospital, Berlin, and was conducted in accordance with the Helsinki Declaration.

### Design

This study aimed to explain effects of tablet use on the clinical routine that were observed in a previous study. It is beyond the scope of this paper to provide full details regarding the study design (please refer to [19]). In brief summary, the study was conducted within groups with one interventional and one control condition. The intervention consisted of tablets equipped with EMRs that were used in addition to the existing information system. The latter comprised a conventional paper chart and a ward trolley with a laptop running a desktop version of the EMR. We employed a crossover design. Tablet use was for a period of 7 weeks in total for all participants. Following the study period, physicians were administered questionnaires. They were instructed to anonymously complete and return the questionnaires within the space of 1 week by dropping them into one of the researcher’s postbox. This was done to ensure full confidentiality of data collection. Five of the devices used in this study electronically collected usage data from participants. Tracking data were automatically captured and subsequently stored on the device.

### Tablets

This study used iPads (iPad mini, Apple Inc, Cupertino, CA, USA) running a mobile EMR (SAP EMR Unwired Version 1.10, SAP AG, Walldorf, Germany). Main features of the software included information regarding ward usage, diagnoses, functional diagnostics, risk factors, laboratory and imaging results, clinical order status, and patient demographics in realtime. Vital signs and medication data remained paper bound in both conditions. Clinical tasks and progress notes could be entered and shared with the backend system. However, physicians were unable to enter clinical orders through the tablet. Although tablets were enabled to connect with the Internet, camera use, the screenshot function, and cloud service use were not available. Physicians were only allowed to install apps if these met data protection requirements as defined by the information technology department. For data protection, patient data were not stored on tablets at any time during the study. Instead, data were saved to a backend that could be accessed through the tablet’s frontend. To access patient data, users had to enter a six-digit alphanumeric code to deactivate the key lock on the tablet. The key lock switched on automatically when the tablets remained unused for more than 5 minutes. The EMR sessions timed out after 2 minutes. A second password was required to access the software.

Participants’ patterns of use within the EMR environment were electronically tracked and automatically stored through the iOS debug mode. Following the study period, usage records were downloaded from the tablets and stored on a computer. The data on the devices were deleted following data transfer. For full confidentiality, the procedure did not allow for establishing a link between user names and datasets.

### Questionnaires

We constructed a survey questionnaire that aimed to examine users’ perceptions of and attitudes toward tablets with mobile EMRs during the workday. Before administering the questionnaire, content validation was obtained through a preliminary study with three physicians. Following a thorough revision process, the questionnaire was modified for clarity. Physicians who had participated in the preliminary study were excluded from subsequent data collection. The questionnaire contained a total of 57 items including six categorical, five open-ended, and 46 5-point Likert scale questions. To control for data validity, 30 items were reverse scored. We used traditional 5-point Likert scales throughout the questionnaire. Scale levels were designed as follows. For normally scored items, 1 indicated a clear improvement, 2 reflected a moderate improvement, whereas 3 indicated no detectable effects. Accordingly, 4 and 5 reflected a moderate or clear change for the worse, respectively. For reverse-scored items, an inverse order of ratings applied (1=clear change for the worse, 5=clear improvement). Physicians were explicitly asked to respond to questions regarding work processes that involved tablet use and to provide feedback on how the tablets impacted their clinical routine (eg, Location-independent data access improved the doctor-patient interaction. 1=strongly disagree; 2=disagree; 3=neutral; 4=agree; 5=strongly agree).They were also asked to state their attitudes toward the tablets (eg, What is your favorite documentation tool during ward rounds? 1=paper chart; 2=ward trolley with laptop; 3=tablet). Response scales were designed to specifically comprise different dimensions of tablet use, including perceived overall efficiency of the tablet, satisfaction of data retrieval with the tablet, and general satisfaction with the device. The questionnaire also required participants to provide basic demographic data such as age, occupational group, attitude toward novel technologies, and gender. Open-ended questions provided the opportunity for physicians to leave individual comments of a more general nature (eg, Are there any further aspects in the context of tablet use that you would like to comment on?). A copy of the questionnaire is provided in Multimedia Appendix 1.

### Statistics

All quantitative data analyses were carried out using SPSS Statistics version 22 (IBM Corp, Armonk, NY, USA). Confidence intervals for 5-point Likert scales were calculated through a bootstrap resampling procedure. A nonoverlap of 95% confidence intervals between the sample’s mean and the mean value of the response scale indicated a significant finding. Also, a sample’s mean value was, by definition, significantly different from any single value if the single value was not included in the confidence interval. For example, if one used a 5-point Likert response scale with the response options 1=much better, 2=better, 3=neutral, 4=worse, and 5=much worse to test whether graphical content was better accessible through tablets than via common computers, then any confidence interval with a lower and an upper margin below the mean value of the scale “3” would indicate a significant finding (eg, 95% CI 2.23-2.84) in favor of the tablet. However, any overlap with the mean value (eg, 95% CI 2.81-3.42) would indicate a nonsignificant finding, whereas any confidence interval with a lower and an upper margin greater than 3 would significantly indicate that graphical content was more conveniently accessed through common PCs. We chose this specific procedure because bootstrapping provides more detailed statistical information than *P* values [22]. Limited space required a careful selection of which questionnaire items to include in this section. Thus, we decided to only include significant findings at this point. Means are reported along with the upper and lower margin of their 95% confidence interval. Reverse-scored items are indicated as such.

We further ran a multivariate analysis to identify factors that predicted physicians’ satisfaction with the tablets. Outcome variables used in this context were “preferred way of documentation,” “motivation regarding future tablet use,” “did the tablet pose a useful extension to the current gold standard,” and “overall satisfaction with the tablet.”

Tracking data were analyzed using Matlab (MATLAB 2008b, Mathworks, Natick, MA, USA). Tablet functions were automatically pooled in categories (eg, reports from diagnostic procedures such as ultrasound and electrophysiology were categorized as “documents”). We then analyzed the access frequencies for each category.

## Results

### Survey Questionnaire

**Figure 1:**
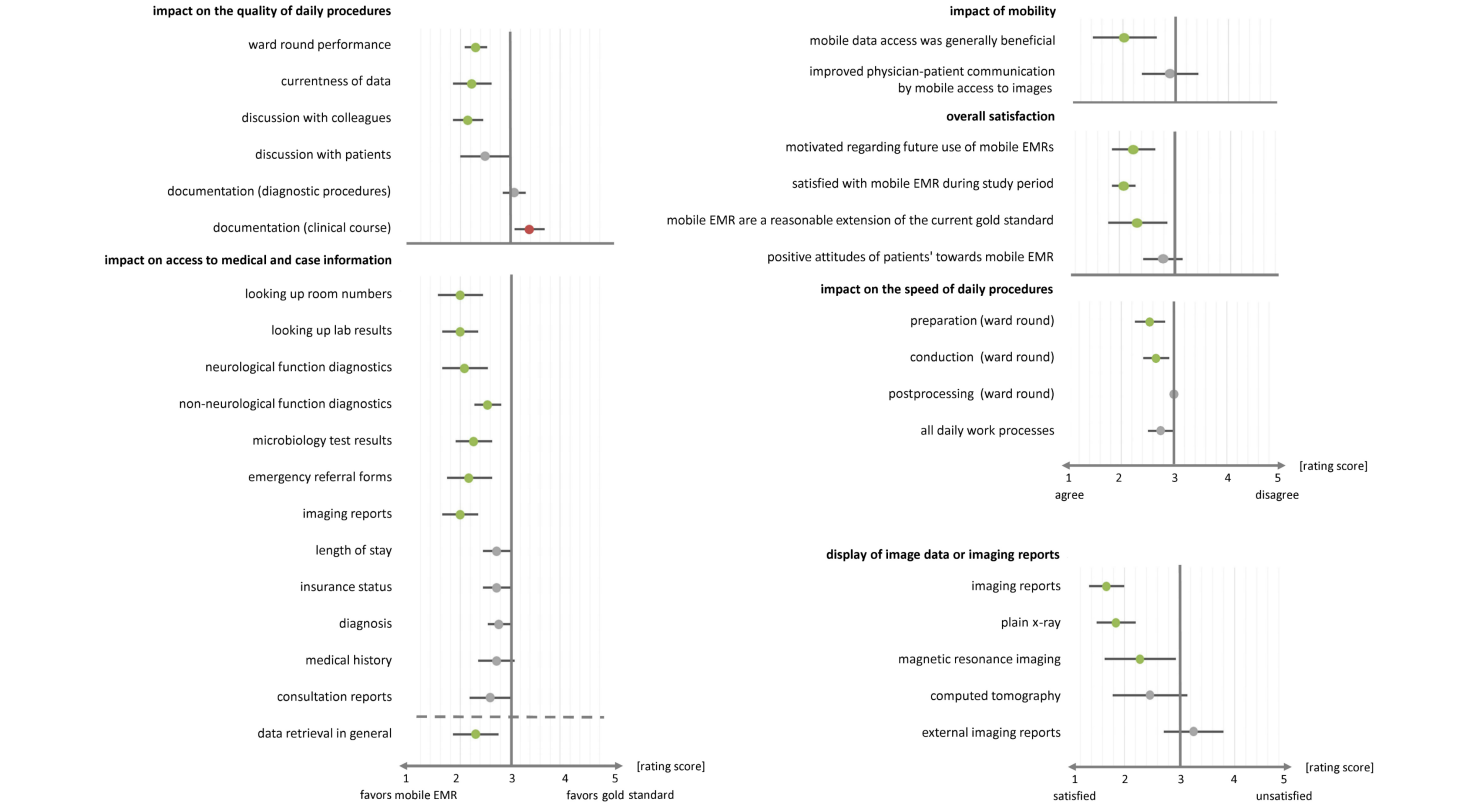
Summary of the survey data representing mean values of participants’ responses (dots) along with their 95% confidence intervals (bars). Colored dots: statistically significant at α=.05; gray dots: nonsignificant result; green dots: improvement through tablet use; red dots: no improvement through tablet use.

All participants completed and returned the questionnaires, resulting in a total of 14 datasets that were included in the final analysis (see Figure 1 for a graphical summary of results). Bootstrapping indicated that participants perceived data retrieval to be accelerated through tablet use (mean 2.31, 95% CI 1.85-2.77). More specifically, they felt that looking up patient’s room numbers (mean 4.00, 95% CI 3.55-4.45, reverse ordered), laboratory results (mean 4.00, 95% CI 3.64-4.36, reverse ordered), neurological function diagnostics (mean 3.91, 95% CI 3.45-4.36, reverse ordered), microbiology (mean 3.73, 95% CI 3.36-4.09, reverse ordered), emergency department referral forms (mean 3.82, 95% CI 3.36-4.27, reverse ordered), and other clinical evidence (mean 3.45, 95% CI 3.18-3.72, reverse ordered) required less time when using a tablet.

The retrieval of graphical material also received high ratings throughout the questionnaire. According to the participants’ responses, x-rays (mean 4.00, 95% CI 3.64-4.36, reverse ordered), computed tomography images (mean 4.00, 95% CI 3.64-4.36, reverse ordered), and magnetic resonance imaging (MRI) images (mean 4.00, 95% CI 3.64-4.36, reverse ordered) were all accessed quicker through tablets. Physicians further reported high degrees of satisfaction with the way radiological evidence was presented on the tablet (mean 4.34, 95% CI 4.00-4.64, reverse ordered). Both x-rays (mean 4.14, 95% CI 3.79-4.50, reverse ordered) and MRI images (mean 3.79, 95% CI 3.07-4.36, reverse ordered) received significantly positive ratings. Tablet representation of clinical evidence without image data was also perceived as positive (mean 3.71, 95% CI 3.14-4.21, reverse ordered). Participants further felt that preparing (mean 2.26, 95% CI 2.31-2.85) and conducting (mean 2.69, 95% CI 2.46-2.92) ward rounds required less time with a tablet. They also stated that tablet use streamlined clinical workflow when carrying out ward rounds (mean 2.31, 95% CI 2.08-2.54). Furthermore, physicians reported improvements when discussing clinical evidence with colleagues (mean 2.23, 95% CI 1.85-2.62) and patients (mean 2.15, 95% CI 1.85-2.46) due to tablet use. Another perceived benefit of the tablet consisted in location-independent up-to-date access to patient data (mean 4.00, 95% CI 3.36-4.57, reverse ordered). Results further indicated that tablet use was generally viewed as a useful extension of the current gold standard (mean 2.29, 95% CI 1.79-2.86). Physicians showed high degrees of motivation to use tablets during future ward rounds (mean 2.21, 95% CI 1.86-2.64).

#### Subjective Time Savings

Average estimates of the tablet’s effect on time required to carry out clinical work processes were as follows. Participants felt that time savings during preparing (4.8 minutes), conducting (6.5 minutes), and postprocessing ward rounds (0.8 minutes) were achieved through tablet use. They further estimated that data retrieval via tablet saved approximately 9.6 minutes per workday.

#### Open Questions

Six participants stated that the opportunity to enter clinical orders directly through the tablet would pose a desirable additional feature. Yet tablets in our study did not provide this function. Five participants stated that discharge reports should be accessible via tablet. Two participants criticized the time-consuming log-in procedure. Further comments included a positive feedback regarding tablet size and the suggestion to make the camera function available for clinical use (eg, to monitor muscle atrophy).

#### Further Items

The question: “What documentation tool(s) do you prefer during ward rounds?” yielded the following responses: trolley with laptop plus tablet (n=2), tablet (n=3), paper chart plus trolley with laptop (n=2), and trolley with laptop (n=1). One participant stated that he or she had no clear preference, whereas two participants did not answer the question.

### Predictors for Tablet Use

Demographic data showed 10 participants were interested in novel technologies, whereas four participants stated that they were not concerned with novel technologies. A multivariate analysis yielded that interest in novel technologies (*F*_1,11_=7.17, *P*=.04) posed a positive predictor regarding participants’ perceptions of the tablet as a useful extension to the established medical information system. The variable occupational group (*F*_1,11_=10.54, *P*=.02) was also a significant predictor in this context with residents rating the device more positively than consultants. The variable age (*P*=.21) did not significantly impact on the outcome variable “did the tablet pose a useful extension to the current gold standard,” but it was a predictor of motivation regarding further use (*F*_3,11_=3.54, *P*=.04) with physicians younger than 40 years of age showing higher motivation. However, none of the other predictors (ie, age, occupational group, interest in novel technologies) were significant regarding the preference for certain documentation tools, motivation for future tablet use, and overall satisfaction with devices.

### Tracking Data

**Figure 2:**
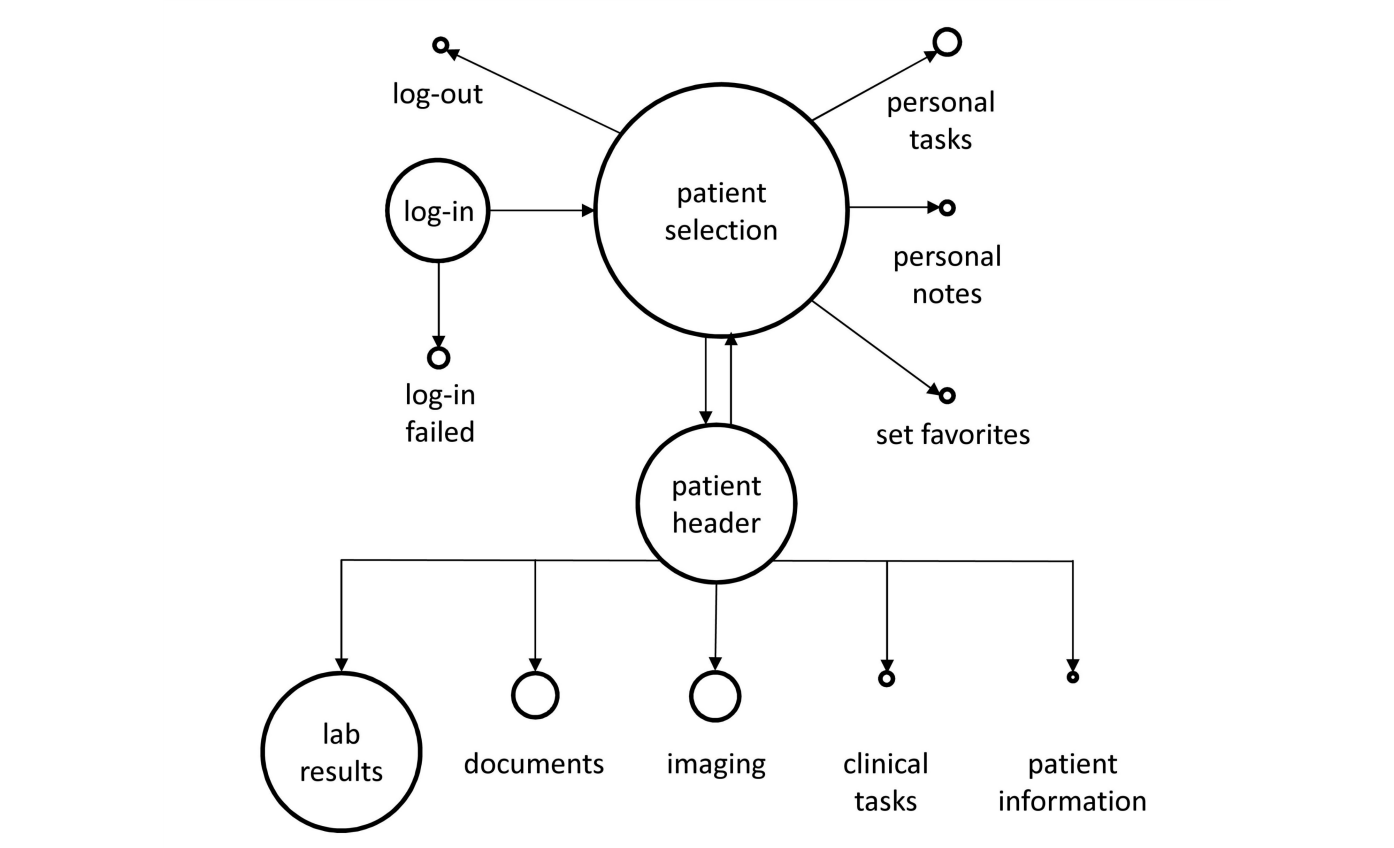
Summary of electronic data tracking illustrating access frequencies of the software features. Only features that made up a minimum of 1% of total tablet use are displayed. Circle size represents proportional access frequencies of software features in relation to one another. Arrows show connections of software features within the electronic medical record menu.

A total of 732 data points were collected through automatic electronic data capturing. Figure 2 displays an overview of the tracking data results. Log-ins failed in 16% (10/62) of the recorded cases. Log-outs were recorded for 6% (3/52) of the sessions only. The remainder of the sessions were logged out automatically after the session had timed out (94%, 49/52). In addition to the navigation, log-in, and log-out procedures, the most frequently accessed features were looking up laboratory results (38.9%, 98/252), imaging data (11.1%, 28/252), and function diagnostics (10.3%, 26/252). Functions that were rarely (<1%) used or not accessed at all included task interaction (eg, sending tasks to a colleague), checking the medical history, and using supplementary features such as drug information and the built-in Web browser. The remainder data points represented subfunctions within functions (eg, use of tools to display trends of laboratory results) and were thus not included in the analysis.

As Figure 2 illustrates, laboratory results, viewing documents, and imaging made up a large share of total use. Interactive tools designed to foster communication among doctors or to create personal notes were used less frequently. Specific software training may encourage users to exploit the full potential of these features. The figure also displays a high number of failed log-ins (approximately 15% of total software access).

## Discussion

The aim of this study was to better understand physicians’ attitudes toward and preferences for tablet use within an inpatient setting. To our best knowledge, this is the first study that investigated physicians’ perception of tablets running an EMR in an inpatient environment and in the context of proven objective benefits [19]. Results indicate high degrees of satisfaction with the devices among physicians along with a strong motivation to use tablets in the future. Participants felt that tablets allowed for quicker access to patient data. They also valued the mobile access to patient data and were contended with the way data were presented on the tablet. In line with the literature (eg, [21]), physicians reported that tablet use improved physician-patient interaction and streamlined clinical workflow. The latter also became evident due to perceived time savings as a result of tablet use. A novel finding from this study was that tablet use also improved interaction between physicians.

Overall results from this study add on to the growing body of evidence indicating that physicians view tablets as clinically useful [16,21,23]. The survey also provides novel evidence for the perceived value of tablets within an inpatient setting. This finding is of great importance because previous studies only examined the issue within emergency departments [21,23] or at a rural practice [16]. However, we also found that novel features of mobile EMRs that extend functionality beyond that of classic paper charts or laptops were rarely exploited.

### Underestimation of Objective Time Savings

In our study, physicians’ positive perception of tablet use resulted largely from the device’s potential to expand and improve access to medical data. One of the main perceived benefits was faster data retrieval through tablet use. This is in line with previous research suggesting that fast access to the required data plays a key role in enhancing physicians’ motivation to use tablets [15]. At this point, however, it is important to note that subjectively perceived time savings in this study were largely congruent with objective time savings that were measured in the context of quantitative evaluations [19]. Although physicians estimated that tablet use saved approximately 10 minutes of time over the entire workday in connection with data retrieval, objective measurements yielded that physicians required approximately 1 minute less for checking a patient’s medical record during ward rounds when using a tablet [19]. Given that physicians on the study ward saw approximately seven patients each during their daily ward round, these estimates appear to be somewhat precise.

However, physicians’ estimates regarding time savings within the context of ward round duration were less accurate. Although they felt that tablet use saved only 4.8 minutes during preparation and 0.8 minutes during the postprocessing of ward rounds, objective time records that were obtained through self-monitoring and direct observation indicated time savings of 20 minutes and 15 minutes, respectively (for full details, see [19]). Similarly, physicians’ average estimate of conducting ward rounds differed substantially from the actual times; participants perceived time savings of 6.5 minutes per ward round, but the time required to carry out ward rounds remained unaffected by tablet use. However, it should be noted that conducting ward rounds was more effective in the presence of a tablet because time savings in the context of checking medical data led to prolonged patient-physician interaction. It remains unclear why physicians were misled in regards to perceived time savings. However, considering the tight schedule of the clinical routine, one obvious conclusion to draw from these results is that perceived time savings played a major role in enhancing physicians’ motivation regarding future tablet use.

### User Satisfaction

Another important insight provided by this study was the high degree of satisfaction among physicians with the way data were presented on the tablet. Previous research suggests that data presentation impacts significantly on physicians’ motivation to use EMRs [15]; however, the previously mentioned finding offers an intuitive explanation about why physicians in this study felt that tablet use improved physician-patient interaction. Previous studies yielded that physicians value the quick and easy way to share medical information with patients as provided by tablets [16,21]. In accordance with these findings, results from our previous study showed that tablet use led to a mean increase of time spent at the bedside of 1 minute per patient encounter [19]. It is plausible that mobile data access encouraged physicians to share medical information over the device with patients, which may explain the increase in time physicians spent at the bedside. This may then have impacted positively on the physician-patient interaction because one determinant in patient satisfaction appears to be the amount of time patients spend with their physician [24].

### Demand for Further Development

However, despite the largely positive feedback in the main aspects of this study, participants also highlighted some limitations of the tablets. In line with previous studies [16], there was a clear demand for additional apps and functions to further enhance the benefit of tablets. Particularly, our sample wanted the opportunity to enter clinical orders through the tablet and a simpler log-in procedure. The latter appears to be of greater practical relevance considering that tracking data revealed a large number of failed log-ins. Fingerprint log-in appears to be a suitable alternative in this context because this would grant an easy, less time-consuming log-in. There were also a large number of failed log-outs, which may spark security concerns regarding clinical tablet use at first sight. However, all participants were instructed to adhere to the strict code of data protection relevant in the context of this research project at all times. They were instructed not to leave tablets unattended at any point and to store devices in their coat pocket when not in use. The fact that the device remained in a safe place when unused provides a plausible explanation why physicians relied on the auto log-out function in the majority of the recorded cases, which made a manual log-out technically redundant. Further limitations are discussed subsequently.

### Exploiting the Full Potential

The software provided a total of 68 functions. However, tracking data yielded that the physicians accessed only 15 functions frequently (more than five times during the study period). These predominantly included looking up medical evidence and especially laboratory results. Accordingly, feedback on this particular dimension was largely positive. The sparse use of tablets for clinical documentation might have been because appropriate functions were not fully available on the devices. The tablet’s software lacked the opportunity to enter clinical orders. Although users could enter personal notes, it was not possible to edit these or to delete outdated notes on the device once they were saved to the system. This could only be done through a desktop workstation. In addition, users might have perceived it as difficult to enter information via a digital keyboard. Therefore, they may have preferred the physical keyboard provided by the laptop that was available throughout the study for a more common keyboard experience. It appears plausible that such shortcomings might have impeded users’ motivation to use the tablet for documentation purposes, leading to a mainly negative perception of the device in this regard. Functions that were also rarely used included the opportunity to create notes and to share these with colleagues. Yet users did not provide feedback on why they neglected these functions. Feedback regarding unused functions was neutral throughout; therefore, one plausible explanation is that participants were unaware of these functions or their potential. Comprehensive training sessions including practical examples might help physicians to explore the full potential of the tablets.

### Limitations

One obvious limitation of this study is the fact that the physicians were free to return to the gold standard whenever they wanted to. Therefore, it cannot be ruled out that tablet use was restricted to occasions when the physicians felt that it would be convenient to use the device. Hence, the study design does not allow us to draw conclusions about whether tablets have the potential to replace the current medical information system. We can only establish that tablets were perceived as a helpful extension to the current gold standard.

Another limitation was the restricted scope of the available functions. In the context of our study, physicians primarily used the tablet as a mobile information system. Thus, it remains unclear how physicians would have reacted to the device if they had the opportunity to enter clinical orders via the tablet.

This study was also limited by the fact that the degree of practical work varied among participants. More specifically, our sample represented a range of user behaviors depending on the occupational group. Therefore, results provide only limited insight into the specific requirements of distinct user subgroups. Finally, this study was conducted at a single institution with a limited number of participants and results from this study might not generalize to other hospitals and other medical disciplines (conservative vs surgical).

### Conclusion and Perspective

This is the first survey providing evidence that physicians perceive the use of tablets as clinically useful within an inpatient setting. These findings may have potentially wide-ranging implications. High degrees of motivation to further use tablets among physicians along with well-documented time savings as a result of tablet use during the clinical routine constitute promising preconditions for further clinical use of the devices. Software improvements and comprehensive training sessions on the device appear necessary to encourage users to exploit the full potential of the tablets. However, to gain a more profound understanding of the tablet’s potential benefits, further studies at different institutions involving health care professionals from disciplines other than neurology will be necessary. In this context, future research should also aim to investigate how physicians respond to tablets as documentation tools.

## Appendix

Multimedia Appendix 1 Survey questionnaire appendix.

## Abbreviations

EMR: electronic medical record
MRI: magnetic resonance imaging
