# Supplementary material for: Mobile Electronic Medical Records Promote Workflow: Physicians’ Perspective From a Survey
Source: JMIR Mhealth Uhealth. 2016 Jun 6;4(2):e70. doi: 10.2196/mhealth.5464 (PMC4914779; doi:10.2196/mhealth.5464)
Supplement: Multimedia Appendix 1 [file mhealth_v4i2e70_app1.pdf]

A. Demographic information

Please tick the appropriate box.

1. Sex

❑ female ❑ male

2. Age group

< 30 years ❑ 30 - 39 years ❑ 40 - 49 years ❑ 50 - 59 years ❑ > 59 years ❑

3. Occupational group

❑ Head physician ❑ Resident ❑ Nurse ❑

4. How many times per week were you involved in the clinical routine during the study period?

❑ < 2x times ❑ < 4x times ❑ > 4x times

5. How would you describe your attitude towards novel technologies?

❑ positive ❑ negative

B. Questions regarding efficiency of the mobile EMR.

B1: Overall efficiency of the mobile information system.

**Using a tablet-PC made...**	Much faster	Somewhat faster	Neutral	Slower	Much slower
...access to patient data...					
...the **preparation** of ward rounds...					

…the **conduction** of ward rounds…					
…the **post-processing** of ward rounds…					
…the entire clinical routine…					

B2. Efficiency of specific sub-processes in the context of data retrieval with the mobile EMR.

**Using a tablet-PC made access to the following data faster/slower:**	Much slower	Slower	Neutral	Faster	Much faster
**1. Patient data**					
Contact details					
Room numbers					
Length of stay					
Insurance status					
**2. Medical evidence**					
Diagnosis					
Medical history					
Clinical Evidence					
Lab Results					
External Lab Results					
Neurological function diagnostic					
Non-neurological function diagnostic					
Microbiology test results					
Emergency referral forms					
**3. Imaging Data**					
X-ray					
MRI					
CT					
External images					
**4. Other**					
Patient care					
Handover between physicians					
Home medication					

Clinical course					

B3. Mobile EMR + goldstandard (Paper chart + trolley laptop) vs. exclusive use of goldstandard

Please provide perceived changes of labour time due to tablet use in minutes.

**Using a tablet-PC changed the amount of time required to…**	Time gain per day **(in minutes)**	Time los per day **(in minutes)**
…carry out ward rounds.	+	-
…prepare ward rounds.	+	-
…post-process ward rounds.	+	-
…retrieve patient data during ward rounds.	+	-

B4. What is your favourite documentation tool during ward rounds? You can opt for up to three answers.

☐ **Paper chart**

☐ **Trolley with laptop**

☐**Tablet-PC**

B5. Are there any further aspects in the context of tablet efficiency that you would like to comment on?

C. Questions regarding satisfaction with the mobile EMR (Quality)

C1. Questions regarding overall satisfaction with the mobile EMR.

**By using the mobile EMR…**	Much better	Better	Unchanged	worse	Much worse

…the process of conducting ward rounds worked…					
…clinical documentation worked…					
…the currentness of clinical evidence was…					
…generating clinical course documentation was…					
…discussing clinical evidence with colleagues / the head physician worked…					
…discussing clinical evidence with patients worked…					
…generating cinical orders worked…					

C2. Questions regarding location independent access of data as provided by the mobile EMR.

	Strongly disagree	Disagree	Neither agree nor disagree	Agree	Strongly agree
I perceived mobile real-time access to patient data generally as enriching .					
Mobile access to image data improved patient-doctor communication during ward rounds.					
I perceived mobile access to the red list as enriching.					

C3. Quality of the mobile EMR with respect to image data.

**How satisfied were you with presentation of data as listed below?**	Very unsatisfied	Somewhat unsatisfied	undecided	Somewaht satisfied	Very satisfied
Radiological evidence					
External radiological evidence					
X-rays					

MRT-images					
CT-images					
Clinical evidence other than image data					

C4. General questions regarding satisfaction with the mobile EMR.

	Very much	Somewhat	Undecided	Not really	Not at all
Have your experiences with the tablet-PC increased your motivation to use a tablet during ward rounds in the future?					
Did patients respond generally positive towards tablet use during ward rounds?					
Did you perceive the tablet-PC as an useful extension of the current goldstandard (Paper chart + trolley with laptop)					
Were you overall satisfied with the tablet-PC as information system during ward rounds?					
